# The Combined Effect of Weaning Stress and Immune Activation during Pig Gestation on Serum Cytokine and Analyte Concentrations

**DOI:** 10.3390/ani11082274

**Published:** 2021-08-01

**Authors:** Haley E. Rymut, Laurie A. Rund, Courtni R. Bolt, Maria B. Villamil, Bruce R. Southey, Rodney W. Johnson, Sandra L. Rodriguez-Zas

**Affiliations:** 1 Department of Animal Sciences, University of Illinois at Urbana-Champaign, Urbana, IL 61801, USA; hrymut2@illinois.edu (H.E.R.); larund@illinois.edu (L.A.R.); bolt2@illinois.edu (C.R.B.); southey@illinois.edu (B.R.S.); rwjohn@illinois.edu (R.W.J.); 2 Department of Crop Sciences, University of Illinois at Urbana-Champaign, Urbana, IL 61801, USA; villamil@illinois.edu; 3 Department of Statistics, University of Illinois at Urbana-Champaign, Urbana, IL 61820, USA; 4 Center for Digital Agriculture, University of Illinois at Urbana-Champaign, Urbana, IL 61801, USA

**Keywords:** bilirubin, calcium, anion gap, interleukin 2, interleukin 1 beta

## Abstract

**Simple Summary:**

The present study of blood chemical, cytokine and hormone indicators in male and female pigs offered insights into the combined effects of maternal immune activation and weaning stress. Our results indicate that maternal immune activation may grant higher tolerance to anion gap and bilirubin disruptions triggered by weaning stress. Therefore, management practices aimed at minimizing the effects of weaning should consider the interacting effect of immune activation during development.

**Abstract:**

Weaning stress can elicit changes in the metabolic, hormone and immune systems of pigs and interact with prolonged disruptions stemming from maternal immune activation (MIA) during gestation. The present study advances the characterization of the combined effects of weaning stress and MIA on blood chemistry, immune and hormone indicators that inform on the health of pigs. Three-week-old female and male offspring of control gilts or gilts infected with the porcine reproductive and respiratory syndrome virus were allocated to weaned or nursed groups. The anion gap and bilirubin profiles suggest that MIA enhances tolerance to the effects of weaning stress. Interleukin 1 beta and interleukin 2 were highest among weaned MIA females, and cortisol was higher among weaned relative to nursed pigs across sexes. Canonical discriminant analysis demonstrated that weaned and nursed pigs have distinct chemistry profiles, whereas MIA and control pigs have distinct cytokine profiles. The results from this study can guide management practices that recognize the effects of the interaction between MIA and weaning stress on the performance and health of pigs.

## 1. Introduction

The impact of weaning on the offspring reflects social, nutritional and environmental stress conditions [1,2]. Weaning stress includes separating the offspring from littermates and sow, handling and transport of pigs, becoming familiar with unknown pigs in the new pen, housing, environment and feeding as demonstrated by studies of rodents and pigs [1,2].

Weaning stress also elicits changes in the metabolic, hormone and immune systems [3,4]. Weaning in the pig can trigger disruptions in intestinal barrier function and permeability. Disruption of the gastrointestinal system can lead to higher translocation of luminal bacteria, toxins and antigens into sub-epithelial tissues. Weaning can impact the liver and kidney function, dysregulating the energy, lipid and protein metabolic processes that can also trigger systemic and chronic inflammatory responses [5]. The previous events can affect the serum level of metabolites, minerals and cytokines. The levels of blood cortisol and lymphocytic trapping in pigs significantly increased the day after weaning [6], whereas the levels of B, T and K lymphocytes decreased in mice after weaning [6].

Maternal influences on animal health and performance can interact with weaning stress. Before weaning, animals can experience other stressors, including prenatal immune challenges resulting from maternal immune activation (MIA). The immune response of females to infection or other insults during gestation can result in fetal exposition to maternal inflammatory and stress signals, impacting fetal development and having long-lasting postnatal effects [7,8,9,10,11,12]. In turn, neurodevelopmental alterations can lead to prolonged disruptions in immune and hormone levels and behavior disorders including schizophrenia and autism spectrum disorder later in life [13,14,15]. The porcine reproductive and respiratory syndrome virus (PRRSV) belongs to the viral order *Nidovirales* that encompasses coronaviruses and can trigger MIA [16,17,18]. Disruptions of respiratory and gastrointestinal function and gastrointenstinal dysbiosis in offspring later in life have been associated with MIA during gestation [19,20,21]. As a result, PRRSV-elicited MIA may hinder the physiology and health of pigs and cause substantial revenue loss in the pig industry [22,23,24].

Persistent behavior and physiological disruptions have been associated with prenatal exposure to inflammation signals in mice and pigs [25,26,27]. For example, immune activation during gestation triggered by the viral mimetic polyinosinic-polycytidylic acid (Poly(I:C)) was associated with significant changes in the levels of cytokine genes in the blood of 7-day-old mice [28]. These changes included the down-regulation of interleukin-1α (IL-1α) and interleukin-12 (IL-12).

Maternal immune activation during gestation can influence the response of the offspring to a second challenge later in life, and this condition is the basis for the two-hit hypothesis [29]. The combined effect of MIA elicited by PRRSV infection and a lipopolysaccharides (LPS) immune challenge of 28-day-old pigs altered the blood levels of IL-1β and IL-10 differently than the accumulation of the individual MIA or LPS challenge effects [15]. Our studies also identified significant effects of the interaction between PRRSV-elicited MIA and immunological and metabolic stressors in the serum levels of cytokines and chemistry and locomotion behavior of 60-day-old pigs [26,27].

Most reports about the two-hit hypothesis have explored the effects of MIA followed by a postnatal challenge such as LPS or Poly(I:C) that is expected to elicit substantial physiological or immunological responses [30,31,32]. We detected significant effects of MIA and a postnatal Poly(I:C) immune challenge in the behavior and blood profiles of 60-day-old pig [26,27]. We propose that the two-hit model is also applicable to the interaction between MIA and less acute postnatal challenges such as weaning. Considering the effect of MIA on neural and immune processes and the effect of weaning on metabolic and immune processes and organ function, both factors could interact in a synergistic or antagonistic manner on energy allocation to the immune, endocrine and other systems. Furthermore, the interacting effects of MIA and weaning may affect feed intake and physiological, and the overall growth and health of the pig. The previous metabolic and inflammatory effects of the double hit from MIA and weaning are expected to be reflected on changes in the blood chemical and inflammatory marker profiles.

The aim of the present study is to further the comprehension of the two-hit hypothesis of weaning and MIA on three-week-old pigs. A comprehensive investigation of the individual and combined effects of PRRSV-elicited MIA, weaning and sex on chemical and immune indicators of organ function and health was undertaken. The conclusions from this investigation can aid in the development of management practices that alleviate the effects of gestational and weaning insults in pig physiology that influence growth and reproductive performance. In addition, the multidimensional profiling and multifactorial experimental design of the present study enable us to offer a comprehensive characterization of the double hit of MIA and weaning on blood biomarkers of pig health and performance.

## 2. Materials and Methods

The experiments in this report were reviewed by the University of Illinois at Urbana-Champaign Institutional Animal Care and Use Committee. The experiments included Camborough 22 gilts (*n* = 17, PIC, Hendersonville, TN, USA) from the University of Illinois at Urbana-Champaign PRRSV-free swine herd. The females were inseminated with PIC 359 semen throughout four replicates spaced approximately 75 days apart. The females were moved on gestation day 69 to containment chambers that house one sow per crate. The gilts had unrestricted access to water and received a diet that supported gestation requirements totaling 2.3 kg/day [27]. Supplementary Table S1 lists the ingredients of the diets used in this study. On gestation day 76, nine gilts were nasally inoculated with the live strain P129-BV PRRSV at a median tissue culture infectious dose equal to 5 mL of 1 × 10^5^ (Purdue University School of Veterinary Medicine, West Lafayette, IN, USA), mixed with Dulbecco’s modified Eagle medium (5 mL total volume). This protocol was proven to trigger PRRSV-induced MIA evidence by decreased feed intake and increased body temperature for 12 days after exposure [17]. The remaining gilts were nasally inoculated with 5 mL of sterile Dulbecco’s modified Eagle medium, and these gilts constituted the control group. The gilts in the control group received the same quantity as the PRRSV-challenged gilts consumed the preceding day to ensure similar energy intake.

Farrowing induction on gestation day 113 to farrow employed 10 mg of Lutalyse (Pfizer, New York, NY, USA), and the offspring stayed with the gilts in the farrowing crates until 21 days of age. Relative to the control gilts, PRRSV-challenged gilts had a lower number of pigs born alive (−2.0 pigs), a higher number of stillborn pigs (0.2 pigs) and a higher number of mummified pigs (0.2 pigs). Among the pigs studied (*n* = 74), approximately half of the pigs in each litter approximately stayed with the gilt, and the rest were weaned at 7:00 AM and housed in groups of 4 to 5 pigs per pen. The body weight of the pigs was not significantly different between MIA groups [17,18], and an equal number of female and male pigs were assigned to the nursed and weaned groups. Gestational exposure to PRRSV was associated with a lower number of pigs born alive, and therefore *n* = 40 control samples and *n* = 34 MIA samples were available. The distribution of pigs among weaning groups was *n* = 35 nursed and *n* = 39 weaned pigs, and the sample size per sex group was balanced. Most MIA-weaning- sex groups had a sample size of *n* = 9 pigs (groups MIA-weaned-male, control-nursed-male and MIA-weaned-female), followed by *n* = 10 (groups control-nursed-female and control-nursed-male), *n* = 8 (groups MIA-nursed-male and MIA-nursed-female) and *n* = 11 (group control-weaned-male). The experimental design is a 2 × 2 × 2 factorial representing the 2 levels of the factors MIA, weaning and sex.

The weaned pigs had unrestricted access to water and received 0.4 kg/pig of a diet that met growth requirements (Supplementary Table S1). Blood collection was at 22 days of age, at 7:00 AM, and after the pigs were intramuscularly anesthetized with a telazol:ketamine:xylazine drug cocktail at a dose of 0.03 mL/kg body weight. The anesthesia cocktail included 50 mg of zolazepam (reconstituted with 2.5 mL ketamine, 100 g/L), 2.5 mL of xylazine (100 g/L) and 50 mg of tiletamine (Fort Dodge Animal Health, Fort Dodge, IA, USA), as per published protocols [17,27]. The ketamine:xylazine anesthetic combination does not affect the blood profiles of non-esterified fatty acids lactate, triglycerides, urea, cholesterol or glucagon in the timespan of the experiments [33]. On the morning of the blood collection, the pigs were not offered food to minimize potential postprandial effects of feeding.

Blood sample collection employed plastic tubes (2 × 9 mL), including clot activator and single-use needles. Blood clotting was attained at room temperature, and centrifugation (1300× *g* at 4 °C for 15 min) was used to isolate the sera that was subsequently aliquoted for chemistry and cytokine panels or cortisol measurement. The serum was stored in a freezer at −80 °C until analyte analysis.

### 2.1. Chemistry Analysis

The selected analytes offer an indication of possible organ activity disruption. In particular, blood urea nitrogen (BUN), creatine-phosphokinase (CPK), aspartate amino transferase (AST) and creatinine can indicate dysregulation of skeletal or cardiac muscle [34]. Bilirubin, BUN, glucose, alkaline phosphatase (AlkPhos), albumin, total protein, the leakage enzymes glutamate dehydrogenase (GLDH) and AST can provide information on changes in liver function [35,36]. Insights into kidney function can be gained from the levels of blood total protein, albumin, creatinine, calcium and phosphorus. The levels of glucose, triglycerides and cholesterol inform on energy balance, while chlorine, sodium, potassium, total protein and bicarbonate are indicators of disorders in the metabolic system and digestive function [37].

Chemistry concentrations were measured with an automated chemistry analyzer (AU5400 Beckman Coulter) that relies on photometric modules (Beckman Coulter, Inc., Atlanta, GA, USA). The within and between-assay coefficients of variation of the assays used to measure the chemical analytes were lesser or equal to 8%. Assessment of chemistry analyte concentrations was performed by the College of Veterinary Medicine Diagnostic Laboratory at the University of Illinois (Urbana, IL, USA), a facility that is certified by the American Association of Veterinary Laboratory Diagnosticians.

### 2.2. Cytokine and Cortisol Analysis

The pig cytokine MILLIPLEX MAP magnetic bead assay (MilliporeSigma, Burlington, MA, USA) was used to profile the blood concentration of 13 cytokines. Profiles were measured at the Bursky Center for Human Immunology and Immunotherapy Programs, Washington University School of Medicine, St. Louis. The multiplex array enabled the measurement of the levels of IL-1α, interleukin 2 (IL-2), interferon gamma (IFN-γ), interleukin 4 (IL-4), interleukin 1 beta (IL-1β), interleukin 6 (IL-6), interleukin 8 (IL-8), interleukin 1 receptor antagonist (IL-1ra), interleukin 10 (IL-10), tumor necrosis factor alpha (TNF-α), interleukin 12 (IL-12), granulocyte-macrophage colony-stimulating factor (GM-CSF) and interleukin 18 (IL-18). Following manufacturers’ instructions [38], processing of the 25 mL of plasma and standards included overnight incubation and wash steps, and duplicate measurements were recorded per cytokine and sample.

The cytokine levels were estimated using the MILLIPLEX Analyst Software v5.1.0.0 (Darmstadt, Germany). Using a best-fit standard curve predicted based on known references [38], cytokine concentrations (ng/mL) were predicted from a five-parameter model [39]. Concentrations from wells with less than 30 beads and cytokines with a coefficient of variation > 30 were filtered. The levels of IL-8 presented had a high coefficient of variation due to the low average level in at least one pig group, and therefore this cytokine was not considered for further analysis. Serum cortisol concentrations were analyzed using an ELISA kit that uses a competitive binding platform according to the manufacturer’s instructions (Cayman Chemical, Ann Arbor, MI, USA).

### 2.3. Statistical Modeling and Testing

Descriptive statistics, including the mean of all pig groups and the baseline group of nursed pigs from control gilts, were computed to compare the observed concentrations relative to previously published standards. In addition, Pearson correlations between the biochemistry and cytokine levels were computed to assess the simultaneous effects of MIA, weaning and sex on the immune and metabolic systems.

Body weight, chemistry, cytokine biomarkers and cortisol observations were described using a mixed-effects linear model. The model accounted for the fixed effects of weaning stress (nursed or weaned pigs), MIA (pigs from PRRSV-challenged and control gilts), sex (female or male pigs), all interactions. Gilt and replicate were included as random effects and accommodated for heteroscedasticity of variance between weaning and MIA levels. The models for blood biomarkers, including the covariate of body weight at day 21, and all models were analyzed using PROC MIXED (SAS/STAT software, Version 9.4, 2019, SAS Institute, Cary, NC, USA) and used the Kenward-Rogers adjustment of degrees of freedom.

Residual distribution tests indicated no departure from Normality assumptions for body weight and cytokine measurements. On the other hand, a natural logarithm transformation was necessary for the residuals of the chemistry and cortisol measurements not to depart from the Normality assumption. The experimental design used permitted the evaluation of three- and two-way interaction effects on the analyte, body weight, and cortisol measurements. A partitioning of the interaction *p*-value using the PROC MIXED/SLICE option enabled to test MIA and weaning effects within the model interaction terms. The patterns of the response variables across MIA, weaning and sex groups were investigated using the least square means (and standard errors) estimates. Only effects that presented an overall *p*-value < 0.05 are highlighted in the Results section and based on this criterion no further adjustments were implemented. The patterns of the significant effects are characterized using the partitioning *p*-value and least square means.

A series of multivariate analyses enabled us to characterize the capability of biochemistry or cytokine concentrations to detect the effects of MIA and weaning. Multivariate statistics including Pillai’s Trace, Wilk’s lambda, Roy’s Greatest Root and Hotelling-Lawley Trace were used to assess the associations between each type of analytes and MIA-weaning groups. One sample had a missing measurement and therefore was not included in the multivariate analysis of biochemistry profiles. In this case, 10 samples had at least one of the duplicate cytokine measurements identified as low based on the five-parameter model used and were not included in the multivariate analysis of the cytokine profiles. The multivariate analysis was implemented using PROC MANOVA (SAS/STAT software, Version 9.4, 2019, SAS Institute, Cary, NC, USA). The multidimensional distance between the four MIA-weaning groups of pigs was tested using the Mahalanobis distance. Finally, the biochemistry and cytokine indicators were combined into canonical index variables using the CANDISC procedure (SAS/STAT software, Version 9.4, 2019, SAS Institute, Cary, NC, USA). Scatter plots of the first two canonical discriminant variables enabled us to visualize the distribution of pigs by MIA and weaning group.

## 3. Results

The impact of weaning stress 58 days after a viral challenge during gestation was evaluated in 22-day-old pigs from both sexes. Descriptive statistics of the chemistry and cytokine concentrations across all MIA-weaning groups of pigs and within the baseline group corresponding to nursed pigs from control gilts are summarized in Supplementary Table S2. The Pearson correlation estimates between the metabolic and immune concentrations among the baseline pigs were consistent in sign, and most were ± 0.14 units from the estimates based on the complete data set across MIA and weaning groups. Therefore, the correlation estimates based on the complete data set are presented.

The most extreme Pearson correlations (r > |0.60|, *p*-value < 0.0001) between chemistry concentrations (excluding analyte ratios and differences that represent part:whole relationships) were detected between chloride and sodium (r = 0.93), creatinine with chloride or sodium (r = 0.72 or 0.63, respectively), glucose and bilirubin (r = −0.75), CPK and AST (r = −0.73), triglycerides with BUN or total protein (r = 0.61 to 0.65) and triglycerides and glucose (r = −0.61). The most extreme cytokine correlations (average r = 0.91) were among the concentrations of IL-2, IL-4, IL-1α, IL-1β and IL-6.

The most extreme correlations between immune and chemistry profiles were identified between IL-12 and globulin (r = 0.62), followed by the correlation between IL-12 and albumin: globulin ratio and total protein. In addition, significant (*p*-value < 0.0001) were the correlations between IL-12 and creatinine or chloride (r = −0.42), IL-1ra and BUN (r = 0.48) and IL-10 and bilirubin (r = 32).

### 3.1. Effect of Maternal Immune Activation, Weaning and Sex on Serum Chemistry and Cortisol Levels

The effects of gestational immune activation (PRRSV-challenged or control gilt) and weaning (nursed or weaned) on the concentration of complementary chemistry analytes were studied on 22-day-old male and female pigs. A summary of the *p*-values of the experimental factors on the concentration of the chemistry analytes, cortisol and body weight are displayed in Table 1. Considering the multiple analytes studied, we focus on patterns supported at the statistically *p*-value < 0.05 threshold.

**Figure 1:**
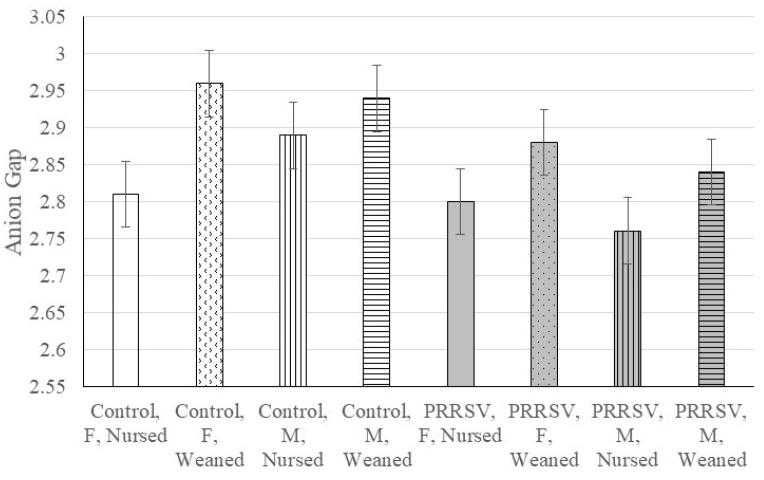
Anion gap (mmol/L, log_e_ units) of the 22-day-old pigs by maternal immune activation (control or PRRSV-challenged gilts), weaning (nursed, weaned) and sex (Female or Male). The horizontal lines depict significant (*p*-value < 0.05) contrasts; whiskers denote the standard error.

**Figure 2:**
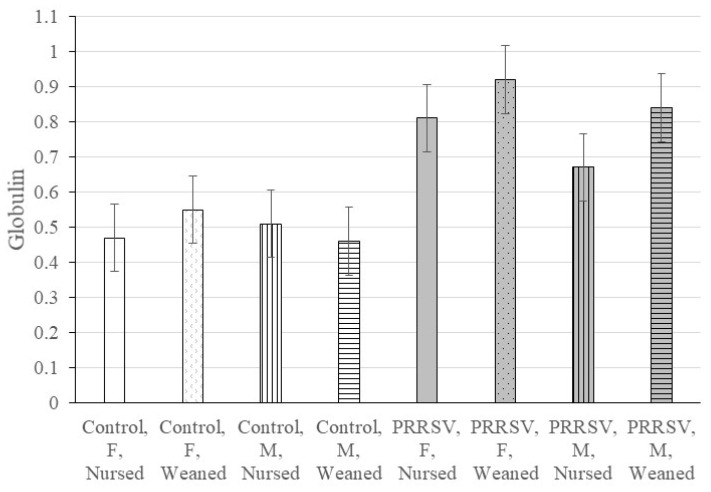
Globulin concentration (g/dL, log_e_ units) of the 22-day-old pigs by maternal immune activation (control or PRRSV-challenged gilts), weaning (nursed, weaned) and sex (Female or Male). The horizontal lines depict significant (*p*-value < 0.05) contrasts; whiskers denote the standard error.

Three and two-way interaction effects (*p*-value < 0.05) were detected for several blood biomarkers. In addition, the *p*-values of the second and first order model terms provide insight into the driving effects in a significant three-way interaction. Therefore, a further breakdown of the interaction effects by MIA, weaning and sex groups are summarized in Table 2 and Table 3. Table 4 summarizes the concentrations of chemistry analyte, cortisol and body weight by weaning, MIA (pigs from control or PRRSV-challenged gilts) and sex groups. Complementing this information, the profile of anion gap is illustrated in Figure 1, and the profile of globulin is illustrated in Figure 2.

Anion gap had a significant three-way interaction between MIA, weaning and sex (*p*-value < 0.027, Table 1), and a significant weaning effect (*p*-value < 0.011). Figure 1 depicts the profiles of anion gap across MIA-weaning-sex groups. The interaction effect was characterized by higher anion gap in weaned relative to nursed pigs (Table 4), and this difference was more marked in females from control gilts (*p*-value < 0.026, Table 2). The anion gap components bicarbonate and potassium also presented significant three-way interaction effects (Table 1) characterized by lower bicarbonate and potassium in weaned relative to nursed females from control gilts (Table 4).

The change in bicarbonate dominated the difference in anion gap, such that nursed male pigs from PRRSV-treated gilts have higher levels of bicarbonate and lower levels of sodium and potassium than all other pig groups.

The concentration of bilirubin had a significant MIA-weaning-sex interaction (*p*-value < 0.018) and weaning (*p*-value < 0.01) effects (Table 1). The bilirubin level was different between MIA groups and between weaned and nursed pigs from control gilts (*p*-value < 0.027, Table 2). Overall, bilirubin levels were lower in pigs from PRRSV-challenged compared to control gilts, particularly among nursed females (Table 4).

The concentration of total protein (including globulin and albumin) exhibited a significant three-way interaction (*p*-value < 0.028) and a significant MIA *p*-value < 0.045) effects (Table 1). The previous effects were characterized by differences between PRRSV-challenged and control gilts among weaned males (*p*-value < 0.019, Table 3) and among weaned females (*p*-value < 0.028, Table 3). The total protein concentration was higher in pigs from PRRSV-challenged compared to control gilts, and among these, weaned pigs had the highest levels (Table 4). The MIA effect on globulin was characterized by significantly higher levels in pigs from PRRSV-challenged than control gilts, and among these, weaned pigs and particularly among weaned females (Table 4). Figure 2 depicts the trends of globulin concentration among MIA, weaning and sex groups.

The concentration of calcium concentrations had significant MIA (*p*-value < 0.033, Table 1) and weaning (*p*-value < 0.016, Table 1) effects). The calcium levels were higher in nursed relative to weaned pigs and particularly low among weaned males from PRRSV-challenged gilts (Table 4).

The levels of CPK presented a significant MIA-by-sex (*p*-value < 0.037, Table 1) and a significant MIA effect (*p*-value < 0.034, Table 1). The interaction is characterized by highest and lowest enzymatic levels detected in females from control and PRRSV-challenged gilts, respectively (*p*-value < 0.005, Table 3), whereas the levels remained similar among males (Table 4).

The effect of weaning on the concentration of cortisol was significant (*p*-value < 0.01, Table 1), and this effect also interacted with sex and MIA (*p*-value < 0.05, Table 1). These effects were characterized by higher levels of cortisol in weaned relative to nursed pigs (Table 4). In addition, the effect of weaning on cortisol was less pronounced in pigs from PRRSV-challenged compared to control gilts and in males relative to females.

### 3.2. Effect of Maternal Immune Activation, Weaning and Sex on Serum Immune Parameter Levels

The fluctuations in the serum concentrations of pro- and anti-inflammatory cytokines in response to MIA, weaning and sex were studied in 22-day-old pigs. The statistical significance of the interaction and main effects on the cytokine concentrations is presented in Table 5. Table 6 lists the cytokine concentrations (estimates and standard errors) by weaning, MIA and sex groups.

The interaction between MIA and weaning had a significant (*p*-value < 0.05) association with several cytokine patterns (Table 5). However, the MIA-by-weaning interaction pattern varied across cytokines (Table 6). The distinct MIA-by-weaning interaction effect observed for the pro-inflammatory cytokines IL-1β, IL-2 and IL-18 was characterized by the highest concentrations observed in weaned pigs from PRRSV-challenged gilts and the lowest among nursed pigs from control gilts (Table 6). Conversely, the interaction pattern detected for the anti-inflammatory cytokine IL-4 was characterized by lowest concentrations in weaned pigs from control gilts and highest among weaned pigs from PRRSV-challenged gilts (Table 6).

### 3.3. Discriminant Analysis of the Effects of Maternal Immune Activation, Weaning and Sex on Serum Chemistry and Immune Parameters

**Figure 3:**
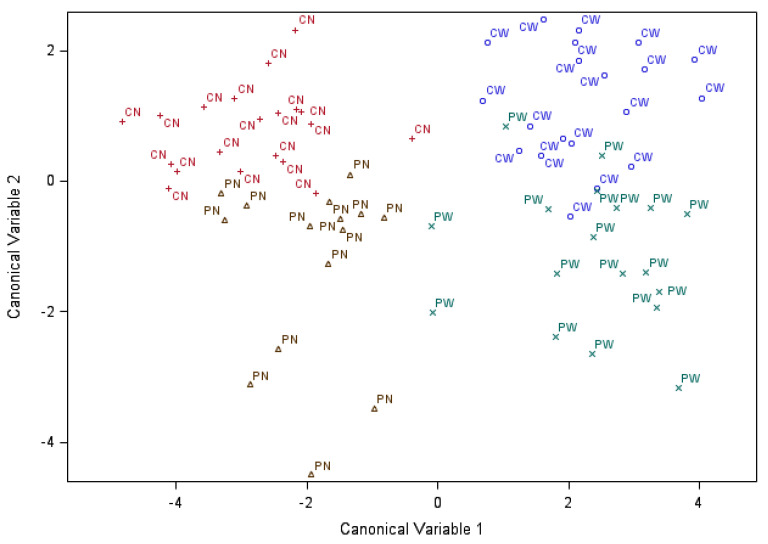
Scatter plot of the pigs labeled by maternal immune activation followed by weaning group (PN = PRRSV-challenged and nursed, PW = PRRSV-challenged and weaned, CN = control and nursed and CW = control and weaned), across the first two canonical discriminant variables using chemistry analyte information.

**Figure 4:**
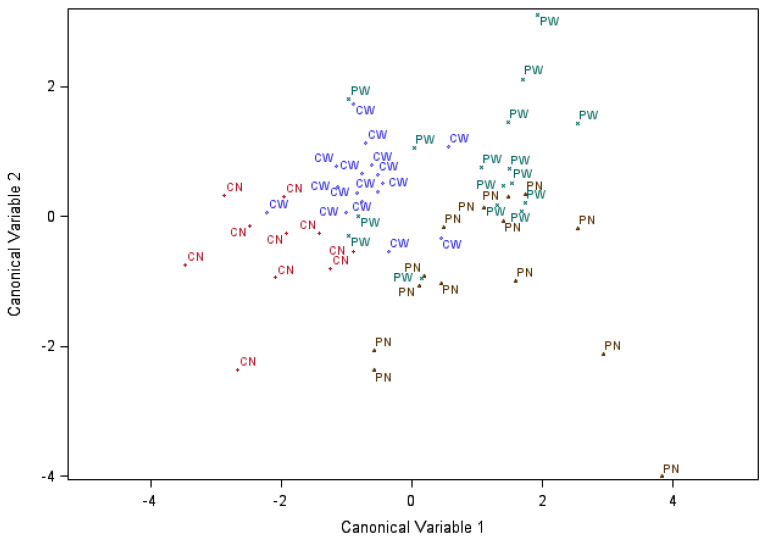
Scatter plot of the pigs labeled by maternal immune activation followed by weaning group (PN = PRRSV-challenged and nursed, PW = PRRSV-challenged and weaned, CN = control and nursed and CW = control and weaned), across the first two canonical discriminant variables using cytokine information.

The results from the canonical discriminant analysis of the samples based on biochemistry analyte or cytokine concentrations annotated by MIA and weaning group are presented in Figure 3 and Figure 4. The first two canonical discriminant variables explained 78.3% and 16.6% of the variation of the chemistry profiles and had a significant association with the MIA-weaning effect (*p*-value < 0.0001 and *p*-value < 0.01, respectively). The combined MIA and weaning effects detected by the chemistry multivariate statistics Hotelling–Lawley trace, Pillai’s trace, Wilks Lambda and Roy’s Greatest Root were significant at *p*-value < 0.0001. In addition, the Mahalanobis distances between all four MIA-weaning pig groups based on chemistry profiles were significant at *p*-value < 0.05.

The first two canonical discriminant variables explained 68.8% and 26.4% of the variation of the cytokine profiles and had a significant association with the MIA-weaning effect (*p*-value < 0.0001 and *p*-value < 0.09, respectively). The combined MIA and weaning effects detected by the cytokine multivariate statistics Hotelling–Lawley trace, Pillai’s trace, Wilks Lambda and Roy’s Greatest Root were significant at 0.0003 < *p*-value < 0.0001. The Mahalanobis distances between all four MIA-weaning pig groups based on chemistry profiles were significant at *p*-value < 0.05, except the distance between nursed and weaned pigs from control gilts (*p*-value < 0.11).

Figure 3 and Figure 4 facilitate the visualization of the double-hit hypothesis both in biochemical and immune profiles. The differentiation of MIA-weaning groups based on the chemistry analytes studied is more notable than the differentiation based on the cytokines studied. The combination of MIA and weaning stress is almost entirely identified by the two canonical discriminant variables for biochemistry analytes, with less than 6 pigs misclassified and less than 15 pigs in the cytokine data.

## 4. Discussion

The dietary and social changes encompassed by the practice of weaning elicit immune and metabolic stresses in livestock. These stresses lead to changes in blood chemistry parameters and stress and inflammation markers [40,41]. Similarly, prenatal MIA can elicit immune stress and prolonged inflammation, and changes in blood cytokine levels [15]. The simultaneous impact of distinct stressors on the immune and chemistry markers is likely to be non-additive because inflammatory conditions can alter organ function and produced metabolites, and conversely, alterations in organ function can trigger immune responses [42]. Thus, we offer evidence of both interacting and independent effects of viral-induced MIA and weaning stress on panels of blood chemistry and cytokine indicators.

The levels of chemistry analytes were consistent with reports and reference intervals previously reported [40,41]. The mean albumin:globulin ratio and concentrations of albumin, AST, creatinine, GGT, protein, urea, potassium and sodium (Supplementary Table S2) were consistent with observed levels in 21, 28 and 35 days-day old pigs (0, 7 and 14 days post-weaning, respectively) [40] and 30 day-old pigs (2 days post-weaning) [41]. The concentrations of glucose and triglycerides were on average higher, albeit within the range of the descriptive data, than the provided reference interval [40,41], and the levels have been attributed to the dietary transition from high fat content, milk based to post-weaning feed that has lower digestibility [40]. The concentration of cortisol was in agreement with the blood levels detected in 14-day old pigs from PRRSV-challenged and control gilts [15], in 21 day-old pigs at weaning [40] and other studies of peri-weaning pigs [43,44,45]. Likewise, the levels of cytokines TNF-α, IL-10 and IL-1β were consistent with serum concentrations in weaned 14-day-old pigs from PRRSV-challenged and control gilts [15]. The average body weight at day 21 was consistent with previous work [46].

The study of the correlation among the biochemistry analyte profiles provided insights into the simultaneous effect of the pre- and post-natal stressors studied on various physiological systems. The importance of sodium and chloride is associated with the role of these minerals in pH modulation, the balance of electrolytes and nutrient absorption [47].

The relationships between creatinine and sodium (r = 0.63) and glucose and bilirubin (r = −0.75) estimated in the present study are consistent with associations among these indicators detected in 130-kg barrows receiving different diets [48]. The significant and positive correlation between sodium and chloride (r = 0.93) observed in the present study agrees with a previous investigation of mineral content in the blood of pigs [49]. Other correlations investigated in the present study have not been commonly reported in pigs but are consistent with patterns in other species. A negative correlation between serum glucose and bilirubin was also detected in humans [50]. The negative correlation between glucose and triglycerides observed in this study may be associated with changes in the energy sources used as pigs mature and are weaned. In agreement with the trends detected in the present experiments, the serum levels of BUN and total protein had a positive association in weaned pigs at the beginning of the growing period across diets with different levels of lysine [51].

Consistent with the known connectivity of the signaling pathways that respond to immune challenges, the concentrations of various cytokines including IL-1α, IL-1β, IL-2, IL-4 and IL-6 were positively correlated. Previous pig studies have reported similar positive correlations among the concentrations of the studied cytokines. A positive association between the serum levels of IL-6 and IL-4, stronger in suckling than in weaned pigs, was detected in a study of immunomodulation of neonatal immunity [52]. The positive correlation between IL-2 and IL-4 has been attributed to the pleiotropic association between these cytokines [53]. IL-2 is a major cytokine responsible for regulating cell-mediated immune responses, and IL-4 participates in T-cell development and differentiation during an immune challenge.

Whereas many studies reported on the fundamental function of IL-6 to elicit immune response to viral challenges, a relationship between IL-6 and worsening of viral disease has been noted in particular conditions [54]. The positive association between IL-6 and other cytokines detected in the present study indicates that IL-6 aids in the development of immune responses to MIA and weaning. The immune challenge elicited by LPS triggered consistent IL-6 and IL-1β profiles in blood monocytes of swine [55]. Likewise, consistent profiles were observed in blood IL-1, IL-2 and IL-6 in response to a Salmonella challenge and yeast supplementation in 21-day old weaned pigs [56].

The covariation between blood cytokines and chemical analytes across MIA, weaning and sex groups highlighted the interdependence between the inflammatory and metabolic systems. High correlations were detected between IL-12 and globulin, albumin: globulin ratio and total protein. In addition, significant were the correlations between IL-10 and bilirubin, IL-12 and creatinine, and IL-1ra and BUN. A positive association between the blood levels of IL-10, IL-12, globulin: albumin ratio, and several globulin fractions including alpha2-, beta- and gamma- was observed in piglets challenged with *Actinobacillus pleuropneumoniae* [57]. Similarly, a positive association between the blood levels of IL-10, IL-1α, IL-6 and of BUN, creatinine, total protein and bilirubin were reported in 3–4 month-old pigs diagnosed with Porcine dermatitis and nephropathy syndrome relative to control pigs [58].

Following the evaluation of chemistry and cytokine basic metrics, we proceeded to test for independent and interacting effects of MIA, weaning and sex on the analyte profiles. A review of MIA and weaning effects is presented in Section 4.1 and Section 4.2 for chemistry and cytokine levels, respectively.

### 4.1. Maternal Immune Activation, Weaning and Sex Effects on Serum Chemistry and Cortisol Levels

Multiple biomarkers presented significant MIA effects, yet the magnitude of the weaning effect tended to be higher than that of MIA. This trend is related to the sampling timing, which was within 24 h of weaning, while MIA was elicited 70 days from sampling. In addition, many serum biochemistry parameters that are biomarkers of renal and hepatic malfunction and tissue dystrophy [59] presented MIA, weaning and sex effects. Disturbances in the systems regulated by renal and hepatic activity can hinder health, growth and reproduction functions.

The anion gap is an index of the difference between the positively charged electrolytes (sodium and potassium cations) and the negatively charged electrolytes (chloride and bicarbonate anions). The interaction between MIA, weaning and sex had a significant effect on the levels of anion gap, and the components potassium and bicarbonate (Table 4, Figure 1). Weaning was associated with higher anion gap levels and potentially higher risk for metabolic acidosis [60] relative to nursing. The previous difference was more prevalent among females from control gilts than other pig groups. Bicarbonate and potassium levels presented a reverse trajectory with lower levels in weaned pigs. The changes in anion gap levels observed in the present study suggest changes in electrolyte balance that previous studies have related to changes in metabolism [60] or dehydration. Increases in anion gap have been associated with renal retention of anions in connection with dehydration in a study of young pigs exposed to *Escherichia coli* [61]. The changes detected in the present study can indicate alkalosis (low anion gap in nursed pigs) or acidosis (high anion gap in weaned pigs) associated with the effect of stressors on liver and kidney function. These findings are also consistent with changes in anion gap in pigs exposed to the stress of transportation relative to control [60]. Except for nursed female from control gilts, the levels of anion gap were lower in pigs from PRRSV-challenged gilts, suggesting that MIA may increase the resilience of pigs to weaning stress.

The effects of MIA, weaning and sex on bilirubin were characterized by decreased levels among nursed females and pigs from PRRSV-challenged gilts. This finding is consistent with the lower serum total bilirubin in 12-kg weaned pigs before experiencing transportation stress [60]. The effect of weaning on bilirubin levels was significant among pigs from control gilts (*p*-value < 0.05, Table 2), suggesting that MIA may increase the tolerance of pigs of either sex to subsequent stress from weaning and support the two-hit hypothesis.

The interaction effects on cholesterol profiles (*p*-value < 0.02, Table 1) were characterized by higher concentrations in females relative to males, in nursed relative to weaned pigs, and these differences were marked in pigs from PRRSV-challenged gilts (Table 4). Creatinine profiles were also characterized by higher levels in nursed relative to weaned pigs and marked group differences among PRRSV-challenged pigs. The patterns detected for cholesterol and creatinine are consistent with the hypercholeterolaemia, hypercreatinaemia, hyperazotaemia (urea) and hyperglycaemia observed in pigs from a farm that had high immune challenge due to elevated bacterial endotoxin and air microbial flora levels relative to a farm with lower levels [62,63].

The increased levels of globulin and total protein in pigs from PRRSV-challenged gilts and weaned pigs are consistent with the higher concentrations of gamma globulin in 5-week-old pigs weaned at 3 weeks of age than continuously nursed during the same period [64]. Consistent with our findings of higher total protein concentrations in weaned pigs, significantly higher levels of acute-phase proteins including amyloid A and haptoglobin were detected in post-weaned calves compared to their pre-weaning levels [65]. In addition, low-dose LPS injection elicited changes in the blood levels of C-reactive protein and serum amyloid A in 1–2 month-old pigs [66,67]. This result prompted the proposal that environmental stressors can modulate physiological control systems that play a role in immunological events. Therefore, these stresses can disrupt the resistance of the animal to infections.

The significant effects of exposure to PRRSV during gestation and weaning on the serum concentration of calcium are consistent with inflammatory or metabolic stress and MIA effects detected on the serum calcium of 60-day-old pigs [27]. In the present study, the level of calcium was lower among pigs from PRRSV-challenged relative to control gilts and among weaned relative to nursed pigs (Table 4). The low calcium levels in weaned pigs is consistent with the lower concentrations of calcium detected in weaned pigs after transportation for five hours [60]. The lowest levels of calcium observed in weaned pigs from PRRSV-challenged gilts and the impact of both hits may predispose pigs to subclinical metabolic disorders that can induce hypocalcaemia [68]. The previous findings suggest that additional calcium availability may be needed to address the needs of pigs exposed to two hits.

The lower CPK concentration in pigs from PRRSV-challenged gilts was more marked in weaned females. Similarly, 60-day-old pigs from PRRSV-challenged gilts exposed to fasting stress had low CPK concentrations; however, the effect was more marked in males [27]. Our finding on 22-day-old pigs is consistent with the use of CPK to assess handling stresses in pigs and reports that the sound level of squealing pigs in an abattoir, an indication of acute stress, was highly correlated with CPK measurements [1].

Weaned pigs had higher cortisol levels than nursed pigs. The significantly higher level of cortisol in weaned relative to nursed pigs is consistent with multiple reports of the multifactorial stressing nature of weaning on pigs [40] and 30 day-old pigs, 2 days post-weaning [41]. In addition, the cortisol concentrations in serum were higher in weaned pigs experiencing transportation stress relative to control pigs [60]. Similar to the findings from the present study, PRRSV-elicited MIA alone was not associated with significant differences in the cortisol levels of 14-day old weaned pigs [15]. A low-grade inflammation triggered by low-dose LPS resulted in changes in cortisol levels in 1 to 2-month-old pigs [60,67]. Likewise, the effect of sex alone was not a significant driver of cortisol concentrations, in agreement with previous reports [69]. The experimental design in our study enabled us to uncover interactions between weaning with MIA and sex, in that pigs from PRRSV-challenged gilts and males presented diminished effect of weaning on cortisol compared to pigs from control gilts and females. An overview of the chemistry profiles indicates that MIA and weaning stresses can have an interacting effect on some blood biochemistry biomarkers. In general, immune activation during gestation appeared to minimize the effect of weaning.

### 4.2. Maternal Immune Activation, Weaning and Sex Effects on Serum Immune Parameter Levels

The patterns of the pro-inflammatory cytokines IL-1β and IL-2 included the highest concentrations in weaned pigs from PRRSV-challenged gilts and the lowest concentrations among nursed pigs from control gilts (Table 6). The higher level of IL-2 in offspring from PRRSV-challenged gilts compared to offspring from control gilts agrees with the higher levels of these cytokines detected in 70-day-old offspring from rats injected with the immune-stimulant LPS during gestation [70].

The pattern of the anti-inflammatory IL-4 encompassed the highest levels among weaned pigs from PRRSV-challenged gilts, but the lowest levels were detected in weaned pigs from control gilts (Table 6). Thus, altogether, the effects of MIA and weaning had a synergistic effect across cytokine function, with the activation of pro- and anti-inflammatory cytokines among weaned pigs exposed to immune activation during gestation.

Weaning may elicit an acute phase immune response through the modulation of inflammatory cytokines. The high levels of IL-1β detected in weaned pigs (Table 6) are consistent with reports of weaning effects in calves. One day after weaning, the level of IL-1β increased in calves relative to pre-weaning levels [65,71]. The level of IL-1β also increased post-weaning in a study of goats receiving milk replacer [72]. Postnatal exposure to PRRSV also triggers cytokine response, increasing the serum concentrations of IL-1β and IL-10 relative to control in weaned three-week-old pigs [73]. In addition, the higher levels of IL-1β detected in pigs from PRRSV-challenged gilts compared to control gilts agree with the higher serum levels of this cytokine in adult rats from dams exposed treated with LPS during gestation [74].

The joint consideration of serum chemistry (Figure 3) or immune (Figure 4) biomarkers using discriminant analysis demonstrated that both factors, MIA and weaning, have systemic effects, as evidenced by the separation of pig groups based on the first two canonical discriminant variables. The chemistry analyte patterns emphasized the separation between weaned and nursed pigs (relative to MIA groups), while the cytokine profiles emphasized the separation between pigs from PRRSV-challenged or control gilts (relative to weaning groups). The discriminant trends may suggest that the preponderance of weaning effects are on organ function that directly impacts blood metabolite and minerals levels. On the other hand, the long-lasting effects of immune activation during gestation dominate the cytokine levels in the blood.

The percentage of the variation of the traits explained by the first two canonical variables was similar between the chemistry and cytokine biomarkers. Likewise, the range of canonical discriminant coefficients was similar between both types of biomarkers, suggesting that both types of biomarkers offer comparable information to discriminate the pigs. However, the chemistry profiles provided higher relative distances between MIA-weaning groups than the cytokine profiles. The minor difference in discrimination strength between profile types may be due to the lower number of biomarkers and higher covariation of profiles offered by the panel of cytokines studied. The consideration of additional cytokines and a higher balance between pro- and anti-inflammatory cytokines may further strengthen the discrimination profile of the immune panel.

The present study offers evidence of the capacity of chemistry and cytokine profiles to discriminate groups of pigs with different gestation exposure to immune activation and weaning stress. This result indicates that chemistry and cytokine profiles have two applications, a) to enhance the understanding of the impact of stresses on the health and physiology of pigs and develop preventive practices and b) as effective tools to identify health and physiological-compromised pigs and implement remedial management practices.

## 5. Conclusions

The understanding of maternal influences elicited by immune activation during gestation and of weaning stress on the health and organ function of pigs was advanced by the simultaneous study of blood chemistry and cytokine profiles. The detection of interactions between MIA and weaning effects offered insights into the modified reaction to weaning stress at 22 days of age associated with immune activation during gestation. Chemistry analyte profiles including anion gap and bilirubin presented an antagonistic interaction effect, such that the effect of weaning was dimmed in pigs from PRRSV-challenged relative to control gilts. Cytokine profiles including IL-1β and IL-2 presented a synergistic interaction effect, such that the effect of weaning was magnified in females from PRRSV-challenged relative to control gilts. Other biochemical biomarkers of liver, muscular and kidney function that presented MIA and weaning effect included globulin, CPK, anion gap, calcium, IL-18 and IL-4.

Canonical discriminant analysis of chemistry and cytokine profiles offered evidence that MIA and weaning have systemic effects on the physiology of pigs. Chemistry analyte patterns enhanced the distinction between weaned and nursed pigs, while cytokine patterns enhanced the distinction between MIA groups. The relative preponderance of each type of analyte panel advanced the understanding of the impact of both types of stress on the pig metabolic and immune systems.

The profiled serum biomarkers can assist in developing and evaluating management practices to reduce the effects of MIA and weaning stress on the pig’s health and advance the understanding of the physiological impact of the stressors. The use of serum markers to guide practices that minimize adverse effects of MIA and weaning requires the simultaneous consideration of chemical and inflammatory markers to address the interconnected nature of the metabolic and immune systems.

## Figures and Tables

**Table 1:** *p*-values of maternal immune activation (PRRSV-challenged or control gilt), weaning (nursed or weaned) and sex (main effects and interactions) on 22-day-old pig chemistry analyte and cortisol concentration, and body weight.

Analyte ^1^	Maternal Immune Activation	Sex	Weaning	Activation-Sex	Activation-Weaning	Weaning-Sex	Activation-Weaning-Sex
AG Ratio	0.053	0.609	0.666	0.355	0.040	0.801	0.175
Albumin ^a^	0.553	0.810	0.169	0.335	0.223	0.757	0.059
AlkPhos ^b^	0.110	0.397	0.804	0.808	0.214	0.657	0.599
AnionGap ^c^	0.075	0.573	0.011	0.151	0.962	0.393	0.027
AST ^b^	0.094	0.542	0.282	0.492	0.112	0.707	0.651
Bicarbonate ^c^	0.136	0.885	0.083	0.634	0.433	0.711	0.045
Bilirubin ^d^	0.548	0.514	0.011	0.361	0.246	0.433	0.018
BUN ^d^	0.216	0.566	0.072	0.377	0.022	0.431	0.082
Calcium ^d^	0.033	0.195	0.016	0.425	0.514	0.562	0.060
Chloride ^c^	0.020	0.820	0.084	0.899	0.936	0.533	0.028
Cholesterol ^d^	0.040	0.159	0.602	0.307	0.102	0.937	0.020
CPK ^b^	0.034	0.988	0.230	0.037	0.151	0.795	0.082
Creatinine ^d^	0.019	0.827	0.102	0.215	0.726	0.791	0.018
GGT ^b^	0.897	0.830	0.015	0.415	0.665	0.490	0.257
GLDH ^b^	0.547	0.444	0.697	0.707	0.521	0.363	0.254
Globulin ^e^	0.025	0.211	0.227	0.329	0.334	0.486	0.080
Glucose ^d^	0.869	0.388	0.002	0.412	0.289	0.830	0.277
NaK Ratio	0.131	0.437	0.151	0.268	0.835	0.112	0.522
Phosphorus ^d^	0.811	0.308	0.159	0.821	0.737	0.473	0.065
Potassium ^c^	0.332	0.052	0.215	0.280	0.922	0.148	0.036
Protein ^a^	0.045	0.556	0.170	0.289	0.804	0.948	0.028
Sodium ^c^	0.028	0.626	0.416	0.723	0.948	0.483	0.061
Triglycerides ^d^	0.197	0.705	0.004	0.707	0.777	0.283	0.085
Cortisol ^e^	0.627	0.201	0.010	0.146	0.261	0.048	0.048
BodyWeight ^f^	0.857	0.439	0.057	0.097	0.341	0.942	0.224

^1^ AG Ratio: albumin:globulin ratio; AlkPhos: alkaline phosphatase; AST: aspartate amino transferase; BUN: blood urea nitrogen; CPK: creatine phosphokinase; GGT: gamma glutamyl transferase; GLDH: glutamate dehydrogenase; NaK Ratio: sodium:potassium ratio; Protein: total protein. Units: ^a^ Log_e_(g/dL), ^b^ Log_e_ (U/L), ^c^ Log_e_ (mmol/L), ^d^ Log_e_ (mg/dL), ^e^ Lo_e_g(ng/mL), ^f^ kg.

**Table 2:** *p*-values of the effects of weaning (nursed or weaned) by maternal immune activation (control or PRRSV-challenged gilt), and sex on the chemistry analyte and cortisol concentrations, and body weight of 22-day-old pigs.

Analyte ^1^	Control Female	PRRSV Female	Control Male	PRRSV Male	Control	PRRSV	Female	Male
AG Ratio	0.594	0.620	0.099	0.126	0.126	0.148	0.716	0.982
Albumin	0.013	0.720	0.065	0.847	0.008	0.530	0.071	0.178
AlkPhos	0.632	0.387	0.631	0.490	0.457	0.330	0.799	0.911
Anion Gap	0.026	0.107	0.214	0.080	0.058	0.034	0.023	0.090
AST	0.384	0.892	0.786	0.542	0.339	0.197	0.824	0.204
Bicarbonate	0.055	0.104	0.048	0.161	0.007	0.042	0.013	0.020
Bilirubin	0.053	0.284	0.027	0.870	0.897	0.039	0.077	0.673
BUN	0.008	0.473	0.176	0.570	0.009	0.407	0.202	0.871
Calcium	0.168	0.150	0.035	0.020	0.271	0.003	0.041	0.145
Chloride	0.372	0.092	0.031	0.107	0.011	0.008	0.015	0.005
Cholesterol	0.437	0.487	0.346	0.651	0.139	0.455	0.274	0.221
CPK	0.065	0.682	0.385	0.888	0.059	0.763	0.375	0.564
Creatinine	0.045	0.110	0.143	0.044	0.139	0.093	0.113	0.117
GGT	0.276	0.554	0.093	0.063	0.052	0.064	0.158	0.021
GLDH	0.610	0.141	0.709	0.759	0.662	0.222	0.169	0.839
Globulin	0.276	0.328	0.550	0.090	0.881	0.140	0.166	0.594
Glucose	0.753	0.109	0.835	0.235	0.950	0.337	0.361	0.299
NaK Ratio	0.453	0.745	0.258	0.348	0.798	0.974	0.408	0.069
Phosphorus	0.013	0.137	0.013	0.035	0.002	0.309	0.124	0.086
Potassium	0.515	0.969	0.064	0.191	0.502	0.292	0.822	0.027
Protein	0.075	0.034	0.459	0.084	0.264	0.433	0.316	0.369
Sodium	0.123	0.065	0.021	0.056	0.270	0.227	0.241	0.558
Triglycerides	0.012	0.144	0.300	0.359	0.011	0.086	0.006	0.168
Cortisol	0.006	0.097	0.174	0.958	0.002	0.149	0.003	0.321
Body Weight	0.273	0.093	0.237	0.137	0.619	0.069	0.201	0.251

^1^ AG Ratio: albumin:globulin ratio; ALP: alkaline phosphatase; AST: aspartate amino transferase; BUN: blood urea nitrogen; CPK: creatine- phosphokinase; GGT: gamma glutamyl transferase; GLDH: glutamate dehydrogenase; Protein: total protein; NaK Ratio: sodium: potassium ratio.

**Table 3:** *p*-values of the effects of gestational immune activation (control or PRRSV-challenged gilt) by weaning group (nursed or weaned), and sex on the chemistry analyte and cortisol concentrations and body weights of 22-day-old pigs.

Analyte ^1^	Female Nursed	Female Weaned	Male Nursed	Male Weaned	Nursed	Weaned	Male	Female
AG Ratio	0.105	0.031	0.640	0.022	0.246	0.017	0.119	0.039
Albumin	0.941	0.224	0.451	0.447	0.898	0.266	0.999	0.324
AlkPhos	0.180	0.407	0.221	0.144	0.136	0.336	0.073	0.127
Anion Gap	0.887	0.224	0.021	0.091	0.146	0.154	0.038	0.424
AST	0.457	0.717	0.486	0.163	0.362	0.095	0.502	0.693
Bicarbonate	0.442	0.097	0.125	0.075	0.186	0.063	0.073	0.160
Bilirubin	0.615	0.206	0.381	0.148	0.289	0.634	0.195	0.704
BUN	0.071	0.903	0.232	0.845	0.079	0.842	0.480	0.209
Calcium	0.484	0.435	0.162	0.250	0.109	0.187	0.233	0.123
Chloride	0.035	0.241	0.213	0.082	0.052	0.049	0.006	0.137
Cholesterol	0.300	0.675	0.086	0.716	0.044	0.323	0.624	0.196
CPK	0.088	0.005	0.930	0.539	0.312	0.012	0.578	0.005
Creatinine	0.521	0.706	0.028	0.221	0.062	0.180	0.012	0.450
GGT	0.781	0.466	0.754	0.708	0.407	0.706	0.308	0.861
GLDH	0.847	0.348	0.979	0.510	0.898	0.267	0.723	0.447
Globulin	0.028	0.022	0.262	0.017	0.076	0.016	0.057	0.017
Glucose	0.222	0.073	0.391	0.751	0.403	0.191	0.672	0.476
NaK Ratio	0.796	0.999	0.208	0.259	0.504	0.565	0.095	0.942
Phosphorus	0.505	0.555	0.285	0.840	0.648	0.322	0.525	0.586
Potassium	0.673	0.695	0.201	0.126	0.567	0.417	0.132	0.973
Protein	0.071	0.028	0.064	0.019	0.167	0.296	0.054	0.640
Sodium	0.153	0.229	0.281	0.090	0.145	0.098	0.029	0.063
Triglycerides	0.152	0.377	0.387	0.394	0.130	0.247	0.256	0.126
Cortisol	0.787	0.360	0.138	0.983	0.436	0.461	0.329	0.422
Body Weight	0.781	0.755	0.990	0.261	0.321	0.968	0.908	0.114

^1^ AG Ratio: albumin:globulin ratio; AlkPhos: alkaline phosphatase; AST: aspartate amino transferase; BUN: blood urea nitrogen; CPK: creatine phosphokinase; GGT: gamma glutamyl transferase; GLDH: glutamate dehydrogenase; NaK Ratio: sodium:potassium ratio; Protein: total protein.

**Table 4:** Chemical analyte and cortisol concentrations and body weight of 22-day-old pigs by maternal immune activation (PRR = PRRSV-challenged or Con = control gilt), weaning (Nur = nursed or Wea = weaned) and sex (Fem = female or Mal = male) group.

Analyte ^1^	Con Fem Nur	Con Fem Wea	Con Mal Nur	Con Mal Wea	PRR Fem Nur	PRR Fem Wea	PRR Mal Nur	PRR Mal Wea	SE ^3^
AG Ratio ^2^	0.55 ^ab^	0.60 ^ab^	0.49 ^bc^	0.65 ^a^	0.26 ^d^	0.19 ^d^	0.41 ^c^	0.22 ^d^	0.07
Albumin ^4^	1.05	1.16	1.04	1.13	1.06	1.08	1.09	1.08	0.04
AlkPhos ^5^	6.73	6.78	6.84	6.79	6.51	6.64	6.65	6.56	0.07
Anion Gap ^6^	2.81 ^abc^	2.96 ^ef^	2.89 ^def^	2.94 ^ef^	2.80 ^ab^	2.88 ^cde^	2.76 ^a^	2.84 ^bcd^	0.04
AST ^5^	3.61	3.94	3.43	3.50	3.84	3.81	3.73	4.02	0.27
Bicarbonate ^6^	3.17 ^bc^	3.08 ^a^	3.15 ^b^	3.09 ^a^	3.22 ^c^	3.17 ^bc^	3.22 ^c^	3.18 ^bc^	0.03
Bilirubin ^7^	−0.96 ^ab^	0.23 ^d^	−1.02 ^ab^	0.07 ^d^	−1.14 ^a^	−0.51 ^c^	−0.70 ^c^	−0.80 ^abc^	0.22
BUN ^7^	1.90 ^a^	2.19 ^bc^	1.92 ^ab^	2.07 ^abc^	2.33 ^c^	2.22 ^c^	2.20 ^c^	2.12 ^abc^	0.14
Calcium ^7^	2.39 ^de^	2.34 ^bcd^	2.43 ^e^	2.33 ^abc^	2.37 ^cd^	2.31 ^ab^	2.37 ^cd^	2.28 ^a^	0.03
Chloride ^6^	4.62 ^cd^	4.63 ^cd^	4.61 ^bc^	4.64 ^d^	4.59 ^a^	4.62 ^cd^	4.60 ^ab^	4.62 ^cd^	0.01
Cholesterol ^7^	5.56 ^d^	5.44 ^cd^	5.30 ^bc^	5.14 ^ab^	5.39 ^cd^	5.51 ^cd^	5.01 ^a^	5.08 ^ab^	0.12
CPK ^5^	6.42 ^c^	6.89 ^d^	6.27 ^bc^	6.50 ^c^	5.94 ^ab^	5.80 ^a^	6.25 ^bc^	6.29 ^bc^	0.18
Creatinine ^7^	−0.51 ^ab^	−0.37 ^ab^	−0.46 ^ab^	−0.36 ^b^	−0.56 ^ab^	−0.40 ^ab^	−0.65 ^a^	−0.46 ^ab^	0.15
GGT ^5^	3.65	3.79	3.63	3.80	3.61	3.69	3.66	3.84	0.08
GLDH ^5^	0.70	0.57	0.45	0.49	0.65	0.38	0.46	0.38	0.18
Globulin ^7^	0.47 ^a^	0.55 ^a^	0.51 ^a^	0.46 ^a^	0.81 ^c^	0.92 ^c^	0.67 ^b^	0.84 ^c^	0.06
Glucose ^6^	4.75	4.82	4.87	4.83	5.13	4.67	5.12	4.80	0.12
NaK Ratio	3.49	3.54	3.56	3.51	3.51	3.54	3.50	3.47	0.04
Phosphorus ^6^	2.26	2.33	2.22	2.29	2.23	2.31	2.17	2.29	0.02
Potassium ^5^	1.42 ^cd^	1.37 ^ab^	1.35 ^a^	1.40 ^bc^	1.39 ^abc^	1.39 ^abc^	1.41 ^bc^	1.45 ^d^	0.02
Protein ^4^	1.53 ^a^	1.58 ^ab^	1.52 ^a^	1.54 ^a^	1.64 ^bcd^	1.71 ^d^	1.63 ^bc^	1.69 ^cd^	0.04
Sodium ^5^	4.92	4.94	4.92	4.94	4.90	4.93	4.90	4.93	0.01
Triglycerides ^6^	3.92 ^a^	4.56 ^cd^	4.13 ^ab^	4.40 ^bc^	4.36 ^bc^	4.84 ^d^	4.39 ^bc^	4.66 ^cd^	0.20
Cortisol ^8^	3.20 ^a^	3.90 ^d^	3.32 ^ab^	3.58 ^bc^	3.15 ^a^	3.61 ^bcd^	3.56 ^bc^	3.57 ^bc^	0.16
Body Weight ^9^	6.35	5.89	5.86	6.13	5.69	6.31	5.45	5.92	0.25

^1^ AG Ratio: albumin:globulin ratio; AlkPhos: alkaline phosphatase; AST: aspartate amino transferase; BUN: blood urea nitrogen; CPK: creatine phosphokinase; GGT: gamma glutamyl transferase; GLDH: glutamate dehydrogenase; NaK Ratio: sodium:potassium ratio; Protein: total protein; Units: ^4^ Log_e_(g/dL)^2^, ^5^ Log_e_ (U/L), ^6^ Log_e_ (mmol/L), ^7^ Log_e_ (mg/dL), ^8^ Lo_e_g(ng/mL), ^9^ kg. ^2^ Least square means estimate. ^3^ Pooled standard error. ^a,b,c,d,e,f^ Differences between groups significant at *p*-value < 0.05.

**Table 5:** *p*-values of the main effects and interactions of maternal immune activation (control or PRRSV-challenged gilt), weaning (nursed or weaned) and sex of the cytokine concentrations on 22-day-old pigs.

Cytokine	Maternal Immune Activation	Sex	Weaning	Activation-Sex	Activation-Weaning	Sex-Weaning	Activation-Weaning-Sex
GM-CSF ^1^	0.512	0.400	0.759	0.136	0.217	0.702	0.679
IFN-γ	0.407	0.025	0.164	0.551	0.055	0.533	0.434
IL-1α	0.463	0.021	0.513	0.096	0.119	0.132	0.419
IL-1β	0.490	0.050	0.440	0.330	0.019	0.071	0.290
IL-1ra	0.505	0.707	0.369	0.791	0.085	0.408	0.725
IL-4	0.511	0.047	0.275	0.201	0.022	0.084	0.339
IL-2	0.642	0.052	0.398	0.195	0.019	0.067	0.337
IL-6	0.911	0.079	0.604	0.503	0.019	0.122	0.411
IL-10	0.494	0.038	0.398	0.147	0.047	0.068	0.285
IL-12	0.481	0.890	0.497	0.273	0.144	0.138	0.161
IL-18	0.452	0.035	0.370	0.209	0.022	0.084	0.315
TNF-α	0.799	0.157	0.423	0.500	0.120	0.777	0.950

^1^ GM-CSF: granulocyte-macrophage colony-stimulating factor; IFN-γ: interferon gamma; IL-1α: interleukin 1 alpha; IL-1β: interleukin 1 beta; IL-2: interleukin 2; IL-4: interleukin 4; IL-6: interleukin 6; IL-1ra: interleukin 1 receptor antagonist; IL-10: interleukin 10; IL-12: interleukin 12; IL-18: interleukin 18; TNF-α: tumor necrosis factor alpha.

**Table 6:** Cytokine concentration (ng/mL) in 22-day-old pigs from by maternal immune activation (control or PRRSV-challenged gilt), weaning (nursed or weaned) and sex group.

Analyte ^1^	Con Fem Nur ^2^	Con Fem Wea	Con Mal Nur	Con Mal Wea	PRR Fem Nur	PRR Fem Wea	PRR Mal Nur	PRR Mal Wea	SE ^3^
GM-CSF	0.19	0.15	0.16	0.12	0.15	0.18	0.22	0.32	0.08
IFN-γ	57.12	38.29	47.31	30.25	60.00	70.78	52.67	47.80	10.33
IL-1α	0.25	0.20	0.25	0.19	0.29	0.44	0.24	0.23	0.06
IL-1β	1.05 ^a^	0.94 ^a^	1.02 ^a^	0.68 ^a^	0.99 ^a^	1.86 ^b^	1.00 ^a^	1.03 ^a^	0.25
IL-1ra	1.32	1.27	1.30	1.13	0.78	1.28	0.92	1.10	0.28
IL-2	1.44 ^a^	1.34 ^a^	1.47 ^a^	0.97 ^a^	1.40 ^a^	2.90 ^b^	1.30 ^a^	1.45 ^a^	0.40
IL-4	6.31 ^a^	5.55 ^a^	6.63 ^a^	3.66 ^a^	5.23 ^a^	12.57 ^b^	4.80 ^a^	5.29 ^a^	1.90
IL-6	0.52 ^ab^	0.44 ^a^	0.5 ^ab^	0.33 ^a^	0.33 ^a^	0.67 ^b^	0.33 ^a^	0.36 ^a^	0.11
IL-10	2.47 ^a^	2.32 ^a^	2.54 ^a^	1.74 ^a^	2.59 ^a^	4.96 ^b^	2.40 ^a^	2.34 ^a^	0.66
IL-12	0.80	0.69	0.74	0.62	0.69	0.89	0.90	0.78	0.10
IL18	4.65 ^a^	4.22 ^a^	4.59 ^a^	3.19 ^a^	4.70 ^a^	8.54 ^b^	4.40 ^a^	4.65 ^a^	1.07
TNF-α	0.65	0.53	0.55	0.39	0.47	0.53	0.44	0.47	0.12

^1^ GM-CSF: granulocyte-macrophage colony-stimulating factor; IFN-g: interferon gamma; IL-1α: interleukin 1 alpha; IL-1β: interleukin 1 beta; IL-2: interleukin 2; IL-4: interleukin 4; IL-10: interleukin 10; IL-12: interleukin 12; IL-18: interleukin 18; TNF-α: tumor necrosis factor alpha. ^2^ Least square means estimate. ^3^ Pooled standard error. ^a,b^ Differences between groups significant at *p*-value < 0.05.

## Data Availability

Data is available upon request from the corresponding author.

## Supplementary Materials

The following are available online at https://www.mdpi.com/article/10.3390/ani11082274/s1, Table S1: Nutrient composition of the diets, Table S2: Descriptive statistics of the serum chemistry (loge-transformed), cortisol, body weight, and cytokine indicators analyzed for all samples and for baseline 22-day-old nursed pigs from control gilts.
