# Supplementary material for: The Combined Effect of Weaning Stress and Immune Activation during Pig Gestation on Serum Cytokine and Analyte Concentrations
Source: Animals (Basel). 2021 Aug 1;11(8):2274. doi: 10.3390/ani11082274 (PMC8388404; doi:10.3390/ani11082274)
Supplement: Supplementary file 1 [file animals-11-02274-s001.zip › animals-1277222-supplementary.pdf]

Table S1. Nutrient composition of the diets.

Ingredients[1] %	Gestation -114 day to 0 day	Lactation 0 day to 21 (weaned) or 22 day (nursed)	Post-weaning day 22
Corn	77.15	67.40	30.74
Soybean meal, 48 % CP	11.55	24.13	20.39
Beet pulp	7.00	0.00	0.00
Blood plasma	0.00	0.00	7.56
Whey, dried	0.00	0.00	25.19
Milk, Lactose	0.00	0.00	10.08
Choice white grease	1.00	5.01	3.02
Limestone	0.75	0.89	1.15
Dicalcium phosphate	1.90	1.86	0.63
Lysine HCL	0.00	0.05	0.04
DL-Met	0.00	0.00	0.14
Salt	0.00	0.00	0.10
Cu sulfate	0.00	0.00	0.00
ZnO	0.00	0.00	0.40
Swine trace minerals premix[2]	0.35	0.35	0.35
Vitamin premix[3]	0.20	0.20	0.20
Sow pac[4]	0.10	0.10	0.00
Total	100.00	100.00	100.00

[1] Diets formulated to meet or exceed the NRC 2012 requirements for swine.

[2] Ingredients: copper, iodine, iron, manganese, selenium, zinc.

[3] Ingredients: vitamin A, vitamin D, vitamin E, vitamin K, niacin, panothenic acid, riboflavin, and vitamin B12

[4] Ingredients: vitamin A, choline, D-biotin, and folic acid.

Table S2. Descriptive statistics of the serum chemistry (log_e-transformed), cortisol, body weight, and cytokine indicators analyzed for all samples and for baseline 22-day-old nursed pigs from control gilts.

	Unit	All Samples		Baseline Samples	
Indicator[2]		Mean	SE	Mean	SE
AGRatio		0.47	0.04	0.45	0.06
Albumin	g/dl	1.08	0.01	1.05	0.02
AlkPhos	U/l	6.72	0.04	6.73	0.04
AnionGap	mmol/l	2.88	0.02	2.82	0.02
AST	U/l	3.75	0.09	3.66	0.12
Bicarbonate	mmol/l	3.16	0.01	3.21	0.01
Bilirubin	mg/dl	-0.34	0.10	-0.96	0.07
BUN	mg/dl	2.16	0.05	2.07	0.06
Calcium	mg/dl	2.35	0.01	2.40	0.01
Chloride	mmol/l	4.61	0.00	4.60	0.01
Cholesterol	mg/dl	5.30	0.04	5.31	0.05
CPK	U/l	6.36	0.08	6.27	0.09
Creatinine	mg/dl	-0.12	0.02	-0.20	0.04
GGT	U/l	3.73	0.03	3.67	0.04
GLDH	U/l	0.35	0.06	0.36	0.11
Globulin	g/dl	0.62	0.04	0.59	0.05
Glucose	mg/dl	4.88	0.03	5.06	0.02
NaKRatio		3.50	0.01	3.52	0.02
Phosphorous	mg/dl	2.24	0.01	2.18	0.02
Potassium	mmol/l	1.42	0.01	1.40	0.02
Protein	g/dl	1.59	0.02	1.56	0.02
Sodium	mmol/l	4.93	0.00	4.92	0.01
Triglycerides	mg/dl	4.43	0.08	4.18	0.11
Cortisol	ng/ml	3.48	0.06	3.26	0.08
Body Weight	kg	6.02	0.14	5.87	0.22
GM-CSF	ng/ml	0.15	0.03	0.13	0.04
IFN-γ	ng/ml	40.87	4.87	36.20	6.07
IL-1α	ng/ml	0.21	0.02	0.18	0.03
Il-1β	ng/ml	0.81	0.09	0.67	0.12
IL-1ra	ng/ml	1.08	0.10	1.02	0.14
IL-2	ng/ml	1.10	0.16	0.79	0.19
IL-4	ng/ml	4.52	0.71	3.05	0.79
IL-4	ng/ml	0.42	0.05	0.38	0.06
IL-10	ng/ml	1.95	0.26	1.49	0.32
IL-12	ng/ml	0.69	0.04	0.73	0.06
IL-18	ng/ml	1.88	0.44	1.10	0.42
TNF-a	ng/ml	0.45	0.05	0.46	0.07

[1]AG Ratio = albumin:globulin ratio; AlkPhos = alkaline phosphatase; AST = aspartate amino transferase; BUN = blood urea nitrogen; CPK = creatine phosphokinase; GGT = gamma glutamyl transferase; GLDH = glutamate dehydrogenase; NaK Ratio = sodium:potassium ratio; Protein = total protein; GM-CSF = granulocyte-macrophage colony-stimulating factor; IFN-γ = interferon gamma; IL-1α = interleukin 1

alpha; IL-1β = interleukin 1 beta; IL-2 = interleukin 2; IL-4 = interleukin 4; IL-6 = interleukin 6; IL-10 = interleukin 10; IL-12 = interleukin 12; IL-18 = interleukin 18; TNF-α = tumor necrosis factor alpha.
